# SUICIDE TRENDS AND DISPARITIES AMONG OLDER ADULTS IN THE UNITED STATES, 2008-2017

**DOI:** 10.1093/geroni/igz038.2550

**Published:** 2019-11-08

**Authors:** Sanae El Ibrahimi, Yunyu Xiao, Matthew L Smith

**Affiliations:** 1 University of Nevada, Las Vegas, Las Vegas, Nevada, United States; 2 New York University, Silver School of Social Work, New York, New York, United States; 3 Center for Population Health and Aging, Texas A&M University, College Station, Texas, United States

## Abstract

Background: Suicide ranks within the top fifteen causes of death among adults 55 and older in the United States and is a growing concern in the face of social isolation and other end-of-life issues. This study examined differences and trends in suicide rates and methods among older adults in the U.S. Methods: Suicide mortality rates from 2008-2017 were derived from the Multiple Cause of Death files in the CDC’s WONDER database. Suicide deaths were identified from the underlying causes of death using ICD-10 codes. Age-adjusted death rates (per 100,000) were calculated. Older adults were grouped into four age categories: 55-64, 65-74, 75-84, and 85+ years. Percent change in suicide rates between 2008-2017 were examined, which were then stratified by gender and top suicide methods. Results: Suicide rates increased by 16% among adults 55 years of age and older from 2008 to 2017 (15.4 vs 17.8 per 100,000 respectively). In 2017, the suicide rate among older adults was 27% higher than the general population (14.0 per 100,000). Suicide rates were significantly higher among men relative to women for those ages 85+ (14:1 ratio of males-to-females). However, females in the 65-74 age group experienced the highest increase of suicide rate (41%) compared to other females or males across age groups. The most common method of suicide was firearms, followed by poisoning and suffocation. Suffocation had the highest increase over time (37%). Conclusion: Rising suicide rates among older adults suggest the need for tailored intervention strategies that address upstream suicide-related risk factors.

